# The Responses of Elite Athletes to Exercise: An All-Day, 24-h Integrative View Is Required!

**DOI:** 10.3389/fphys.2017.00564

**Published:** 2017-08-14

**Authors:** Billy Sperlich, Hans-Christer Holmberg

**Affiliations:** ^1^Integrative and Experimental Exercise Science, Institute for Sport Sciences, University of Würzburg Würzburg, Germany; ^2^Swedish Winter Sports Research Centre, Mid Sweden University Östersund, Sweden; ^3^School of Sport Sciences, UiT The Arctic University of Norway Tromsø, Norway; ^4^School of Kinesiology, University of British Columbia Vancouver, BC, Canada

**Keywords:** wearable sensors, training intensity distribution, monitoring, physiological, athletes, biofeedback

The current research topics in Frontiers of Physiology include “Training intensity, volume and recovery distribution among elite and recreational endurance athletes” (Frontiers in Physiology, 2016) and “Wearable Sensor Technology for Monitoring Training Load and Health in the Athletic Population” (Frontiers in Physiology, 2017). As editors of both of these topics, we would like to share some thoughts concerning (a) how they are fundamentally linked and (b) why we believe it is essential to have an all-day, 24-h integrative view to understand elite athletes' responses to exercise.

Athletes who train frequently each week schedule their training and off-training for days (i.e., microcycles, for example, tapering periods, blocks of training) to as long as months (i.e., macrocycles, for example, periods of preparation with different focuses or training camps) to ensure progressive adaptation and prevent fatigue, boredom, and injury. From this perspective, a fundamental goal is to distribute exercise and off-training effectively over a certain period of time (for example, one or several seasons) to achieve optimal adaptation.

Here, we highlight the importance of an all-day, 24-h integrative perspective on training, emphasizing the fact that conditions outside training significantly modulate adaptation, thereby complicating analysis of the distribution of training intensity.

Elite athletes invest a significant amount of time per year in their training, which in some sports amounts to approximately 17% of their waking time (Fiskerstrand and Seiler, 2004). This means that the remaining 83% is spent on activities such as recovery, including for example, massage, physiotherapy, medical treatments, eating, as well as activities of daily living (including sitting, lying, working, studying, active, and passive transportation) and social engagements (for example, media, sponsor, and family activities). All of these activities modulate psycho-biological responses to training.

The various approaches for improving recovery include massage (Poppendieck et al., 2016), cooling (Poppendieck et al., 2013), stretching and self-myofascial release (Beardsley and Skarabot, 2015), neuromuscular electrical stimulation (Babault et al., 2011), compression attire (Born et al., 2013), active recovery (Laursen and Jenkins, 2002; Buchheit et al., 2009; Riganas et al., 2015), and many more, and most of these modalities are performed for relatively short periods of time (from minutes to perhaps 1–2 h or longer) and usually soon after training. Most of these are designed to improve the delivery of oxygen and substrates to muscles and the clearance of metabolites, thereby attenuating or delaying the onset of muscle soreness and rapidly restoring homeostasis, through regulation of growth and transcription factors (Coffey and Hawley, 2007). Clearly, recovery must be taken into account when evaluating the different responses of elite athletes to exercise.

Since moderate-to-light activity (for example, walking or cycling) enhances muscle blood flow, it is surprising that we can find no studies on the influence of physical activity off-training on the biological and psychological outcomes of exercise, especially since the athletic population is alarmingly sedentary when not training (Weiler et al., 2015).

In addition, other factors such as sleep (Nedelec et al., 2015; Gupta et al., 2017) and nutrition (Thomas et al., 2016) are both influenced by the stress of training and, in turn, modulate the response to training in a significant fashion. In addition, drug abuse by and/or frequent medication of athletes may result in epigenic changes and consequently influence physiological adaptation (Kanherkar et al., 2014). It seems imperative that such factors also be taken into account when comparing different approaches to training.

The busy schedules of (elite) athletes involve a relatively high level of psycho-biological stress, due for example, to frequent traveling for short and long distances, often across time zones (Kölling et al., 2016; Fowler et al., 2017), which detracts from preparedness for subsequent training and competition. More understanding is required here as well.

Moreover, environmental factors, such as exposure to an elevated (Sperlich et al., 2017) or lowered level of oxygen (Girard et al., 2017), variations in temperature (Lorenzo et al., 2010; Kruger et al., 2015), and atmospheric stressors such as ozone, particulate matter (Giles and Koehle, 2014), and ultra-violet radiation, exert an impact on various tissues of the human body and thereby potentially modulate responses to training. Accordingly, such factors should also be considered when judging the responses of elite athletes to exercise.

In addition, psycho-social stress resulting from, for example, media exposure, financial and family concerns, fans, and/or one's own expectations may well influence responses to training.

Thus, it appears virtually impossible to take all of these factors into consideration when studying a homogenous group of elite athletes, not even in a controlled laboratory setting. However, both retro- and prospective analyses on the responses and adaptation to training should provide as much information about such modulators as possible. In this context, we feel that a combination of wearable technology and smartphone-based applications should prove invaluable, since this is the only technology that currently allows as much information as possible to be obtained by continuous 24-h monitoring of, in addition to the internal and external training loads themselves, sleep, traveling, various environmental conditions and psycho-social status. As long as scientific quality is maintained (Duking et al., 2016; Sperlich and Holmberg, 2017) and personal data protected, such technology can potentially provide 24-h feedback (Duking et al., 2017) to the athlete and supporting staff concerning the various psycho-biological responses to training. In this regards, future findings on “Wearable Sensor Technology for Monitoring Training Load and Health in the Athletic Population” (Frontiers in Physiology, 2017) will hopefully help provide innovative approaches to investigating the “Training intensity, volume and recovery distribution among elite and recreational endurance athletes.”

## Author contributions

All authors listed have made a substantial, direct and intellectual contribution to the work, and approved it for publication.

### Conflict of interest statement

The authors declare that the research was conducted in the absence of any commercial or financial relationships that could be construed as a potential conflict of interest.
